# Fitting up the X-Ray Department

**Published:** 1912-10-12

**Authors:** Alfred C. Norman

**Affiliations:** House Surgeon at Durham County Eye Infirmary.


### ELECTRICITY IN MODERN MEDICINE.*

### XIX.—Fitting up the X-Ray Department.

By ALFRED C. NORMAN, M.D. Edin., House Surgeon at Durham County Eye Infirmary.

Having described in some detail the individual pieces of apparatus used in the production of *x*-rays, we now come to consider the fitting up of the installation as a whole, and this is a matter of some importance, for the best instruments obtainable will give but poor results if they cannot be made to work together in perfect harmony and under perfect control.

### The X-Ray Room.

In the choice of a room we must be guided by the amount of work likely to be done and by the amount of space at our disposal. In private practice the *x*-ray installation need not occupy more than a corner of the consulting room, but in hospitals the larger the room the better, for it is important that patients do not come into involuntary contact with wires or instruments, and it is sometimes a difficult matter to prevent such an accident in a small and congested room. In a hospital of from 50 to 100 beds the *x*-ray room should measure at least 15 feet by 20 feet. It should contain one large window provided with a black shutter † so that the room can be made perfectly dark when examinations have to be made with the fluorescent screen. It is an advantage to have the walls and ceiling of the room painted a dead black, as this, by obviating the possibility of reflected light, makes it much easier to obtain complete darkness for screen work. If possible, provision for ventilation should be made through some channel other than the window, for in a climate like ours the latter must be kept shut on many days of the year otherwise the instruments will be ruined by damp air. The *x*-ray room should be lighted by a 100 c.p. metallic filament lamp controlled by two switches, one placed near the door, the other close to the switchboard.

### The Photographic Dark-Room.

Photographic plates are at once spoilt by exposure to white light or to *x*-rays—hence it is necessary to provide a dark-room in which they may be safely handled and developed. This room should be near the *x*-ray room, but should be separated from it by at least a good brick wall, for *x*-rays will easily penetrate a wood partition or door or even lath and plaster. It is a good plan to build the dark-room as an *annexe* of the *x*-ray room, communicating with the latter by a short corridor, and to fix partitions partly across the corridor in a way that will prevent all light from entering the dark-room, and yet will allow of our walking in and out without having to open and close a door. This arrangement, known as a "light-trap," can be fitted up by any builder and is exceedingly convenient, for it enables us to leave the dark-room without risk of admitting white light while plates are being developed.

The dark-room should be at least 10 feet square; it should be painted a dead black inside, and should be provided with the following fixtures: a small window, glazed with two thicknesses of dark ruby glass, for use by day; a ruby lamp, preferably electric, for night work; an ordinary electric light fitting, with a switch distinctive from that of the ruby lamp so that the white light cannot be turned on in mistake for the red; a developing sink provided with hot and cold water if possible; and a shelf or table, about 4 feet long by 2 feet wide, which should be reserved entirely for plate changing, and should be fixed as far as possible from the developing sink in order to avoid contamination by chemicals.

### Supply of Current for the X-Ray Room.

Current used for the *x*-ray work is generally regarded by the electricity companies as "power" current, and in most instances they will, if asked, supply this current at about one-half the price charged for "lighting" current. There is no difference in the current, of course, but it pays the company to create a demand for power current because it is required mostly in the daytime, when the dynamos at the electricity works would otherwise have very little to do.

When an *x*-ray installation has been decided upon, the company should be asked to give a special quotation for the current, and, when this is accepted, they will bring special cables to the *x*-ray room, and will there fix a separate meter and fuses so that the current bill for the *x*-ray department can be kept separate from that for general lighting. For ordinary *x*-ray work an "18-ampere" meter, with cables and fuses to correspond, will be quite large enough; but if instantaneous work is to be done the company should be warned that a specially large meter, capable of carrying 50 amperes or more, will be required.

### The "Overhead System."

Where a continuous current supply is available it is a simple matter to fit up and arrange the apparatus to the best advantage; but when a generator outfit has to be installed it is advisable to have it fitted up by an engineer, and the makers should be asked to send a man for the purpose. The safest and, in the writer's opinion, by far the most satisfactory way of arranging the apparatus is the "overhead system." That is to say, the induction coil, the wires from the secondary coil, and everything connected with them are fixed high up in the *x*-ray room, where they cannot be accidentally touched by patient or operator; and the high tension current is brought down to the *x*-ray tube by two

* Previous articles appeared on Nov. 11, 25, Dec. 9, 30, Jan. 13, 27, Feb. 17, March 9, 30, April 20, May 4, 25, June 8, July 6, Aug. 3, 17, 31, and Sept. 28.

† Not a blind, which would be bound to give trouble sooner or later.

vertical wires that can be avoided easily, even in the dark, for they are situated directly above the tube stand. The switchboard and interrupter are fixed to a wall of the room where they are most easily accessible, while the couch and tube stand occupy the centre of the floor, and can be freely approached from every side so that the operator has absolute freedom in his work. When it is remembered that current from the secondary coil may reach 250,000 volts or even more, the advantage of keeping everything connected with this circuit well out of the patient's reach is obvious, for contact with any part of it would give rise to an exceedingly painful shock. With the aid of the local electrician and a joiner we proceed, then, to fit up our apparatus as follows:—

### Fitting up the Apparatus.

The induction coil should be placed upon a shelf situated about the centre of any wall of the $x$-ray room that has no door or window. This shelf should be about 7 feet from the floor, it should be of hard wood, 4 feet long, 18 inches broad, and 1¼ inch thick, and must be securely fastened to the wall, for the induction coil may weigh as much as 2 cwt.

The switchboard should be bolted securely to the wall directly below the shelf; the centre of the switchboard should be about 5 feet from the floor.

The interrupter should be supported on a small shelf or bracket situated close to the switchboard, either below it or to one side of it.

The main wires at the back of the switchboard must be connected with the main cables (already brought into the room by the power company), and the electrician should be instructed to fix a "double pole" switch on the wall as close to the switchboard as possible in order that the board may be entirely cut off from the main supply whenever it is not in use. A pair of safety fuses should also be inserted at some point between the main cables and the switchboard.

The next step is to connect the switchboard with the coil and interrupter, and this is best done with pieces of insulated flexible wire.* The two terminals on the switchboard labelled "motor" must be connected by wires with the motor terminals of the interrupter. The terminals on the switchboard labelled "coil" must be connected, by a pair of wires, with the primary terminals of the induction coil; but *one of the wires must be cut* and its cut ends connected with the terminals of the interrupter contacts in order that the current shall be *rapidly interrupted before it passes through the coil.* And, lastly, for reasons stated in The Hospital on April 20, page 64, the two *condenser* terminals of the coil must also be connected with opposite contacts of the interrupter.

This completes the fitting up of the various accessories connected with the primary circuit. If current be now sent through this circuit a fresh current will be generated in the secondary coil and will spark across a considerable air-gap between the discharging pillars, thus indicating the enormous voltage we have to deal with in the secondary circuit. This voltage calls for care in fitting up the various accessories in the secondary circuit, for the current has a strong desire to get to earth, and will spark across a space of several inches in order to reach anything capable of conducting it to earth; hence every part of this circuit must be kept well away from the walls and ceiling of the room, from gas brackets, water pipes, and all other metal fittings, and, above all, from electric-light wires and fittings. The wires must be placed where they cannot be accidentally touched, for the human body is an excellent conductor to earth of such high tension current, and wires connected with opposite pillars of the coil must be separated by at least a foot, otherwise sparking would take place between them. How, then, can we best set up our apparatus to fulfil these requirements?

* Of a size technically known as 200-forties.

### Mounting on the Frame.

The writer has tried many plans, and he finds that the most satisfactory is to mount the various accessories on a wood frame suspended in mid-air from a wood rail. The wood rail should be fixed from wall to wall horizontally across the middle of the $x$-ray room; it should be parallel with, and about 10 feet from, the shelf on which the coil stands, and should be about 9 feet 6 inches from the floor. The frame, made of perfectly dry wood and painted black with some non-conducting pigment, consists of two sides and a top. The sides are 2 feet 6 inches long and are about 15 inches apart. The top is fixed by means of screws to the under aspect of the rail in such a way that the sides hang vertically downwards, their free ends being about 7 feet from the floor. The various accessories mounted on the frame are as follows.

At the top of each side there is a terminal for connecting with the positive and negative pillars respectively of the induction coil. The oscilloscope tube and milliampere-metre are fixed on the left-hand side; the valve tube or spark gap, as the case may be, is fixed on the right side; and at the bottom of each side there projects from its free end a terminal for connecting with the cathode and anode respectively of the $x$-ray tube. In addition to the above, metal fittings are provided for the reception of the plate and sliding rod, which constitute the "spintermeter" of the induction coil. This device would be out of our reach if left on the coil, so we mount it on the frame, where we can easily reach it by standing on a chair or foot-stool, for, as we shall see later, it will be frequently required to estimate the hardness of the tube.

(*To be continued.*)

